# The nature of nursing practice in rural and remote areas of Greenland

**DOI:** 10.3402/ijch.v72i0.20964

**Published:** 2013-08-05

**Authors:** Lise Hounsgaard, Anne Birgitte Jensen, Julie Præst Wilche, Ilone Dolmer

**Affiliations:** 1 Institute of Nursing and Health Science, University of Greenland, Nuuk, Greenland and Research Unit of Nursing, Institute of Clinical Research, University of Southern Denmark, Odense, Denmark; 2 Queen Ingrid Hospital, Nuuk, Greenland; 3 Ministry of Health and Infrastructure, Greenland; 4 Institute of Nursing and Health Sciences, University of Greenland, Nuuk, Greenland

**Keywords:** Ethnographic study, Greenland, nursing practice, remote and rural areas

## Abstract

**Background:**

The Greenlandic Healthcare Reform (2010) required improved quality of services for health promotion, prevention of infectious and lifestyle diseases, family nursing and evidence-based clinical nursing.

**Aim:**

To investigate current nursing practice in Greenland and to identify whether it meets the requirements of healthcare reform.

**Design:**

This ethnographic study utilised documentary analysis, participant observation and qualitative interviewing carried out in remote areas of Greenland during 2011–2012. Eight registered nurses, four women and four men, aged between 35 and 55, participated in this study. Four were working at healthcare centres in towns and four were working at nursing stations in villages. The nurses were educated in Greenland or a Nordic country and had been practicing nursing for at least 2 years in an Arctic region. They were observed for 1–5 days, and subsequently interviewed. Interviews included in-depth questioning, based on emerging outcomes from observation. Interviews were recorded and transcribed; they were analysed within a phenomenological–hermeneutic approach.

**Results:**

Nurses in rural and remote areas navigate their health promotion and preventive work with conflict between health strategies and everyday realities, where unpredictable tasks often lead to prioritisation of urgent, acute work. There is interaction between personal and professional skills. Everyday life is characterised by opportunities and challenges in the grey areas, namely nursing, medical and social work.

**Conclusion:**

The nature of nursing practice in rural and remote Greenland is characterised by a high degree of variability and complexity, with a requirement for a wide range of knowledge and skills. Nurses need to be better prepared with regard to acute medical care, preventive care, social work, humanistic approaches and information technology to implement the ideology of health strategies.

The Greenlandic Healthcare Reform places new demands on nurses to carry out diagnosis and treatment of patients, whilst also implementing health promotion and prevention activities (1). The reason for the reform was partly because recruitment of healthcare personnel has become increasingly difficult in Greenland, as in other remote areas (2). Also, it has been necessary to some extent to delegate tasks from physicians to nurses because health monitoring by the Board of Health indicates a need for increased monitoring of residents’ health status with regard to certain conditions, for example, infectious diseases such as tuberculosis (TB) (3). Lifestyle changes impact healthcare, including an increase in smoking-related diseases such as cancer and obesity, which threatens the health of children, adults and older people alike (4, 5). Developments such as early disease detection and prevention of diabetes, cancer and cardiovascular disease may run on separate tracks and must be coordinated to make a difference. The unique opportunities available to local healthcare professionals to gain knowledge of health conditions by way of natural proximity to the local population gives insights into the everyday life of families and those in the workplace (6). This knowledge can be exploited and targeted for intervention in relation to risk factors such as smoking, obesity and inactivity. Generally, there is great need for action to help improve the health status of the people of Greenland (7).

Nurses in rural and remote Arctic areas have a wide range of functions and roles, involving not only traditional nursing in clinical care but also tasks related to medical treatment and social work being devolved to nurses. Moreover, there are many logistical challenges because of the geography and climate of Greenland. There are many challenges for, and expectations of nurses, but there is only limited knowledge of what nurses currently spend their time doing. Therefore, there are many tasks that can potentially be addressed by nurses with the right knowledge and skills. The health reform also opens the possibility to analyse existing nursing practice to get a picture of challenges and problems before implementation of new methods.

The Health reform puts a responsibility on nurses and demands provision of a wider variety of services that are evidence based (8). However, there is also a need to know what their roles demand to make sure that nurses have the right qualifications and training within the Bachelor of Science in Nursing (BScN) and the newly established Diploma in Specialized Arctic Nursing (9).

This article reports a study that investigated nursing practice in rural and remote areas in Greenland. Ethnographic methods were employed to provide insight into the knowledge and experience that is the basis for nursing action, and informs the adaptation and development of local health services.

The aim of this study is to investigate current nursing practice in Greenland to identify whether it meets the requirements of healthcare reform.

## Materials and methods

### Design

Ethnographic methods incorporating documentary analysis, participant observation and interviews (10) have been shown to be a suitable method for nursing research (11) to investigate aspects of everyday nursing practice. Consequently, these methods were incorporated within this design.

### Setting and participants^1^

Nurses in nursing stations and healthcare units were invited to participate on a voluntary basis.^2^ Eight registered nurses (aged 35–55) took part: four women and four men. Four were working at healthcare units and four at nursing stations. The nurses were educated either in Greenland or another Nordic country and had been practising nursing for at least 2 years in an Arctic area. Two nurses were newly qualified and the remainder had been educated several years ago. The nurses had broad and varied professional backgrounds without specific training or competencies in nursing in rural and remote areas. During observation periods, the researcher stayed at the healthcare unit or nursing station shadowing the nurses for about 1–5 days, depending on how the opportunity presented. Entries in nursing charts describing nursing activity were accessed and copied; informed the development of the subsequent interview guide, together with notes taken from the participant observations.

Interviews were conducted at the nurses’ workplaces, in a suitable private room at a convenient time for the participants. Each interview lasted between 45 and 90 minutes.

Of the 8 study participants, 1 nurse was qualified in Greenland, 5 in Denmark, 1 in Sweden and 1 was qualified in the Faroe Islands. One nurse was an intensive care specialist, one a manager of a large nursing home, another had experience of healthcare in an international context, including refugee work, teaching and work for a non-governmental organisation. They were all positive about the high level of autonomy in their jobs, having a great deal of responsibility for performing nursing care in their current roles. They described themselves as mature people with broad life experience.

### Ethical considerations

The study was conducted in accordance with ethical guidelines for nursing research in International Council of Nursing (ICN) (12). Written informed consent was obtained before the study commenced. The Research Ethics Committee for Scientific Health Research in Greenland No. 2012-061467 approved the study.

## Data analysis

A phenomenological hermeneutic interpretative approach was taken for data analysis, based on that described by Ricoeur (13, 14) and further developed by Scandinavian researchers (11, 15, 16).

Material from interviews, nurses’ chart notes and participant observations that were transcribed and analysed. Ricoeur describes the interpretation of a text as an on-going dialectical movement between explanation and understanding. To understand a text is to follow its movement from significance to reference: from what it says to what it talks about. The interpretation method involved three analytical steps: naive reading, structural analysis and in-depth understanding (13). The naive reading aimed to be non-judgmental and to open up insight into the meaning of the text as a whole. A structural analysis was conducted in order to explain the text and to identify and formulate themes. As is apparent from Table I, this step took the form of a movement between units of meaning and units of significance in the text leading towards in-depth understanding.

We have chosen to systematise the total data material in three categories (17): structure, process and outcome. *Structure* refers to the setting in which nursing care is delivered, including appropriate facilities and equipment, logistics, communications, roads, administration and operation of programmes. *Process* relates to matters that deal with how nursing is specifically provided. This includes, for example, relational, communicative and ethical aspects. Processes include dynamics, ethics, attitudes, phenomena, reactions, and so on.

## Results

In a movement between units of meaning (what is said/quotations) and themes (what is being talked about), the structural analysis identified three themes: navigation between health strategies and everyday realities; interaction between sensitivity and rationality; and intervention between opportunities and challenges.

In the following discussion, the three themes are presented separately for clarity (see Table I) but on the understanding that they interact with each other in reality.

**Table I:** Result of structural analysis with categories, units of meaning and themes

Categories	Units of meaning (what is said)	Themes (what is talked about)
Category: Structure	“Yes, well, I’ve not started on prevention work, because I’m still putting out fire …” *(Int.7, female nurse)* “Health promotion has to be served up with something social” *(Int.6, female nurse)* “There’s complex paper communication – often no district doctor – only locum doctors, who don’t know the system” *(Int.5, male nurse)* “… everything I do I write down in Æskulap … I go in for continuity” (*Int.4, female nurse)* “… am a doctor, a lab technician, handy man, shovel snow, collect salt-grit, vaccinate dogs … there’s a lot of social work” (*Int.6, Int.7, female nurses, Int.5, male nurse)*	**Theme 1** Navigation between health strategies and everyday realities Prevention when possible Health promotion through social events Complexity of professional communication Documentation as a possibility Community worker and clinical nurse
Category: Process	“The local residents call me by my name, but also see me as a nurse … The depth of your work depends on how well you know people” *(Int.6, female nurse, Int.8, male nurse)* “What helps is that you know the cultural methods … humility and respect are important” *(Int.8, male nurse)*	**Theme 2** Interaction between sensitivity and rationality Cultural sensitivity in the personal and professional process
Category: Outcome	“It’s hard to distinguish between what constitutes a nursing and a medical action” *(Int.5, male nurse)* “I could really use some knowledge about social work legislation …” *(Int.8, notes, male nurse)* “… there are so many things we do, and roles … you are a part of the community, very close, you have to be really careful to maintain confidentiality” *(Int.7, female nurse)*	**Theme 3** Intervention between opportunities and challenges Carrying out tasks that lie within the medical domain Carrying out tasks that lie within the social work domain Adaptation to necessary roles and functions in the local community

## Category 1: structure

### Theme 1: navigation between health strategies and everyday realities

#### Prevention whenever possible and health promotion tied to social situations

Nurses working in rural and remote areas have insight into strategies for health promotion and prevention of lifestyle diseases and their underlying objectives.

Preventive work is done on a small scale … it should not be transferred into administrative settings and projects … documentation and schemes take you away from the core work. (Int.8:8–9, male nurse)

The nurses take prevention on board in cooperation with the patient and consider that prevention must be integrated in relation to the opportunities available locally. In their view, preventive work should not be led by projects organised far away from their everyday lives. However, diagnosis and treatment have first priority in their work because sudden illness often requires acute intervention, and this can be at the expense of prevention.

Nurses’ talk about putting out fires and unpredictability in their daily work, and a special effort has to be made to keep health promotion and prevention in focus.

The nurse has to ‘serve up’ a social event that is characterized as health-promoting for the idea to kick in within the local population. (Int.6:20, female nurse)

It is necessary to think socially and creatively to implement organisational strategies; for example, when the station nurse chooses to give a course on lifestyle diseases in association with Bingo in the village or when setting up health events to include the whole family. This requires extra effort and ingenuity. It is important to have local people on board as volunteers in any health project, otherwise it can be a very lonely task.

Unlike station nurses, nurses at healthcare units increasingly have opportunities to prioritise targeted health promotion and preventive action:

I think we work very preventively and towards health promotion, including with those who just turn up … so they get an understanding of what these issues involve. (Int.2:7, female nurse)

#### The complexity of professional communication

The study also points to aspects of professional and interdisciplinary communication within this infrastructural context. Nurses make an effort in written communication with partners to support continuity and consistency of patient care. As far as possible, they use the electronic health record, “Æskulap”. However, telemedicine is used only sporadically, despite the fact that telemedicine equipment is available at all nursing stations and healthcare centres. The telemedicine equipment, called “Pipaluk”, registers patient cases that can subsequently be forwarded to the doctor in charge at the nearest regional hospital for diagnosis, supervision, and so on. The reason why it is only used sporadically is that it is an information technology that can only report factual data – not data relating to the patient's psychological or existential situation (fieldwork notes).

#### Opportunities involving documentation

Systematic documentation of the nursing process is a method that is used selectively, that is, as the need arises in each situation, since diagnosis and treatment form the main content of the documentation. The standard pre-printed nursing charts which include problem/diagnosis, intervention and evaluation leaves very little room for additional entries. In addition, some nurses perform double documentation by making preliminary notes which they later transfer into the electronic system (fieldwork notes).

#### Community worker and clinical nurse

One last aspect concerning structural conditions involves a range of non-clinical tasks that nurses perform in the local community.

It involves all kinds of practical work, which has nothing to do with clinical practice … you cannot be too grand to shovel snow in front of the entrance to the nursing station, and when salt-grit has to be put down, you have to collect it and do it yourself. We haven't got a man employed to do it …. We try to take the good with the bad. The longer you are here the more you learn how to get others involved … such as the firemen. (Int.6:9, female nurse)

The nurses also talk about working as secretaries, laboratory technicians, post distributers, porters, and so on. The function seems to constitute a kind of all-round “community worker”.

## Category 2: process

### Interaction between sensitivity and rationality

#### Cultural sensitivity in the professional and personal process

While nurses’ professional and personal skills are challenged, it is relational factors that largely occupy the individual:

It's completely fine that there is a kind of friendship between you and the people … being able to laugh and cry together. (Int.8:.9, male nurse)

Knowing the patient and the family appears to be of great importance for the outcome of nursing. Assessments and interventions are often based on how, on the one hand, the nurse gleans information from families and communities, because she is considered a friend and one of the village's residents with whom everyday life is shared, and on the other hand, how the nurse is the professional in whom one can have confidence at times of illness. This places an indirect requirement on nurses to be role models and examples of good morals and health. Several nurses talked about the importance of respect and humility in relation to earning trust but also of how having knowledge of the patient and family as well as other networks and local knowledge of logistics are pre-requisites for carrying out the kind of nursing that suits the individual's unique situation. As one nurse says:

I always have an open door to one of the boat owners. (Int.8:6, male nurse)

The above a statement that refers to the unpredictability of the small community's need for help at times of illness, and a realisation that anything can happen.

#### To know the cultural methods

The nurse must be aware and perceptive, and use imagination and creativity. Knowing the “cultural practices” can for example mean that you do not plan check-ups and screenings during the different hunting and fishing seasons, as it may be more important for a Greenlander to go on a hooded seal hunt than to be available for a check-up.

The most important thing for the nurse is to understand the culture – to be able to handle the cultural methods, to comfort when sorrows and such things … (Int.6:female nurse)

Several of the nurses highlight the importance of being able to “look behind the surface”; with their local, gradually acquired knowledge, intuition and experience:

You have to look and look and listen and listen to acquire the experience, so you build up a bank of experience … it becomes an experience thing …. (Int.3:7)

## Category 3: outcome

### Intervening between opportunities and challenges

#### Carrying out tasks that lie within the medical domain

The nurses were only partly prepared for the major responsibilities they faced. Particularly in the first 6–12 months, they felt constrained by their lack of knowledge of the field:

It was probably just that there was only me, and of course I could call the hospital, but … it's a question of having to make decisions on lots of things without actually being properly equipped, right? But after a year, I think I got on top of things. (Int.4:7, female nurse)

Nurses point to the diversity of their tasks and functions, whether it is at a hospital, a healthcare unit or a nursing station.

It can sometimes be difficult to distinguish between what constitutes a nursing task and a medical task. We do everything the doctors say you must and can do. We give chemo, blood and medicine intravenously, stitch wounds, set plaster casts, insert catheters, conduct ECG tests, do gynaecological examinations … prescribe Marevan … we do almost everything, so it is difficult to define the limits. (Int.5:1, male nurse)

Those tasks that involve diagnosis or treatment and that are of an instrumental character are said to constitute an essential part of nursing work. It is typically only where there is some doubt that the nurse contacts a colleague to seek advice. In these cases, they prefer to approach the doctor as the closest work colleague, as one can thereby learn medical and surgical procedures that are necessary for everyday work. The contact is usually by e-mail or telephone – it is only possible to arrange medical visits in remote towns and villages a few times a year. It is those occasions in particular that nurses glean as much medical and surgical knowledge as they can. Nurses at nursing stations all express that they perform extended surgical tasks and consider themselves to be treatment nurses, and they pay a lot of attention to their own competence levels.

#### Carrying out tasks that lie within the social domain

In remote areas, it is difficult to separate social work from health-related issues: “TB is not only a health problem – it is also a social problem … accommodation density plays a role … the patient's problem was not that he was at risk of developing tuberculosis bacteria because he kept stopping his treatment, his problem was that he did not have shampoo and soap for daily hygiene” (Int.4:7, female nurse)

Patients’ problems often lie elsewhere than in the ailments they present with. The nurses tell of many psychiatric problems and issues associated with severe disease. One nurse at a healthcare unit trained as a family therapist in order to be qualified to work with patients with suicidal tendencies. Another nurse expresses the need for knowledge of social work:

For my part, I have to admit that it would be more satisfying for me to know about social support measures. (note from a nurse: Int.8)

A nurse tells of an invitation from a patient to participate in “kaffemik” – a Greenlandic “open house” tradition – on his child's birthday. The nurse had previously warned the family that the child could be forcibly removed because of parental alcohol abuse and probable neglect. The parents went through a lot of trouble in arranging the event, and one of the purposes, besides celebrating the child's birthday, was to convince the nurse that their alcohol consumption was reduced and that the patient and his wife were able parents.

Some nurses talk about what they do to make local authorities accountable, by referring social work cases to them, but the local authority system has difficulty in meeting certain tasks. Finally, they also mention that they may act as a career counsellor, that is, a conversation about health could reveal that a young person may need such advice.

#### Adapting to necessary roles and functions in the community

It appears that the nurse dynamically adapts his/her role and function to meet patient needs. The health-structural context of small settlements is dependent on the ability to navigate flexibly between what is stipulated in health policy and the real needs of the local community. It shows up, for example, the way things are communicated and handled when it comes to dealing with a contagious disease, such as TB. In addition to the work in forestalling transmission of the disease, the patient's integrity and identity must be taken into account, and the nurse must ensure that the small community still has confidence in him/her as a person as well as a professional (fieldwork notes).

## Discussion

### Structure elements must keep up with the need for developing arctic nursing

The study explored the nature of nursing practice in rural and remote Greenland. The nurses described themselves as having broad and varied professional backgrounds. None of the participants in the study had specific prior training for practice in rural and remote Greenland. They have to deal with stressful and challenging tasks that are complex and demanding, both professionally and personally. In the absence of programmes for systematic training as a prerequisite for employment, the predominant learning method is “learning by doing”. In a Canadian study, Minore et al. (18) discovered that although skilled nurses are recruited, they are not sufficiently prepared, thus recommending that all nurses who are placed in the Arctic region should have a thorough introduction to the particular nursing context, culture and specific local conditions they can expect to encounter. At nursing stations, where there are no other nursing colleagues, certain skills have to be acquired from a local and often experienced but less qualified healthcare worker. This may cause problems, because such knowledge is not necessarily based on nursing evidence. According to Willman et al. (19), this implies that the basis for action rests on integrating the best available scientific evidence and making decisions in collaboration with the patient. Nevertheless, Nexøe et al. (2) report that nurses felt competent in what they are doing in 76% of the cases.

Health promotion and preventive initiatives that, according to the health care reform, constitute a major focus area are reported to take place “in the margins” – that is, when the pressure of the urgent work eases off. Nor does there appear to be consensus on the focus and direction for action, and it is suggested that it is probably mainly nurses and midwives who take on preventive work. A Canadian study tracked the same trend: “Health promotion and illness prevention activities are displaced by the pressure from acute treatment demands, while follow-up care is often impaired by communication breakdowns attributable to the staffing situation” (18). It looks like almost all Arctic nursing intervention is linked to diagnosis and treatment, leaving little time for health promotion and prevention, whatever the best intention. This can mean that levels of social health inequalities, reflected in smoking, poor self-evaluated health, suicidal thoughts and excess weight are not reduced (7).

### Process elements related to personal role and personal knowledge are principal

The personal process associated with working in a remote and rural area is reported to require not only imagination and action on many fronts but also wisdom, with the ability to see behind situations and people. Personal qualities play an important part in working in rural and remote places, which is reflected in the ability to balance relationships and handle unique patient and family circumstances. Chinn and Kramer (20), suggest that personal values can be expressed as congruence, authenticity and genuineness, including humility and respect for the other person, which are said to have great significance on relationships. This involves processes that relate to personal knowledge that do not arise from rational theory but presuppose the experience of the self as more than rational and intimately connected with others. Cultural sensitivity also comes into play, as it will often be Danish nurses who meet and treat Greenlandic patients. In culture-sensitive communication, it is expected that the nurse has a brave attitude, where he/she looks for similarities in the differences in the interaction with the patient and where he/she must be curious and open (21). A brave attitude can therefore be an essential skill, as some of the nurses also mention it as the importance of knowing “the cultural practices” or as Ruth Lange, who is passionate about the subject, expresses, “to obtain insight into how people are historically, culturally and religiously in the society they live and work in” (22). Eskebjerg (23) concludes that a lack of options does not have to lead to lack of action, but can show the way to the opportunities hiding in non-action. It is about how things look when you consider them in a circular rather than a linear and structured way, when there is status in being humble and not self-serving, and the focus is on the community and the family.

The nurses in the study are aware that they are simultaneously regarded as both local residents and professionals; they appreciate the significance of maintaining friendship with local people despite the occasional personal confrontations and having to take action associated with disease risks or infection. MacLeod et al. (24) maintain that, in small communities, nurses’ personal and professional roles are inseparable. The merging of nurses’ everyday practice and their personal lives must be taken into account when developing policies and service programmes.

### Outcome elements depend on the development conditions for the nursing discipline to integrate interdisciplinary possibilities

In relation to professional process, the clinical view and acquisition of acute-intensive skills needs to be mentioned. Interventions that are of both a medical and surgical character form an essential element of nursing work, as nurses step into the role of the medical specialist (another grey area not without problems). According to Danbjørg (25), the science-based medical approach requires a solid base, which is reflected in the scientifically based procedures and methods in which doctors and specialist nurses are competent. In the absence of programmes for systematic training as a pre-requisite for employment as nurses in Arctic regions, nurses essentially have to seek to extend their knowledge by observing and consulting with doctors. They create, so to speak, a template for clinical work, but they have not developed a causal reasoning for their actions, and their training does not involve on-going systematic professional reflection. What they do cultivate is an experience-based knowledge and an expanded remit in relation to instrumental skills such as stitching wounds, prescribing treatment, X-ray study of fractures, applying plaster casts after prescription, diagnosis and treatment of infections (23).

Social work is another part of the professional process which occupies the nurses, especially when viewed in the context of disease, poverty, violence and abuse. Bergmark (26) shows how social initiatives deal with the interaction between people and their social environment and in particular connect people with systems that can offer help, in terms of resources, services and opportunities. The nurses refer to their own lack of knowledge in this field, where they rarely have interdisciplinary colleagues, yet it is through their “grey” social work that health promotion results can be achieved.

There are difficult conditions for interdisciplinary work in the Arctic region. Nurses in rural and remote areas collaborate with many other professionals – often at great distance and by telemedicine, e-mail and telephone. The nurse appears to function in a sort of buffering role, where she mitigates for the lack of other professional groups being on hand (2). In addition to lacking an interdisciplinary aspect, there are also issues relating to the mono-disciplinary aspect. Each nurse seems to operate within his/her own learning and training. At nursing stations, there is typically only one nurse and a health assistant, whereas nurses at the healthcare unit have colleagues with whom they can share knowledge. The nurses point largely to “learning by doing” and “self-help” as examples of their learning and maintenance of skills; along with their acquisition of knowledge from various doctors and medical staff from agencies that hold consultations in the small towns and villages. The geographical distance also presents an obstacle to meeting with colleagues. In a collection of recommendations, a Canadian study has pointed to the fact that “new ways are needed to systematically design and provide relevant continuing education for rural and remote nurses, including providing education on site, sufficiently supporting nurses to travel for further education, and using information technology. This last mode may require sufficient investment in relevant communication systems and hardware” (24).

## Conclusion

The systematising of data in three categories and the structural analysis used to create themes suggest that the data can be viewed on both an individual and an organisational level. On the individual level, there exists a relation in clinical nursing, where interaction with patients and relatives takes place. The organisational level is about structures, environments and conditions that influence care actions and nursing. Nursing practice in rural and remote Greenland is characterised by high variability and complexity in both levels. Nurses work at a great distance from medical support with meagre levels of information and communication technology. Therefore, nurses need a wide range of knowledge and skills. Their practice involves a mix of nursing, medical and social work. At the same time, the intertwining of nurses’ everyday practice and their personal lives means that their personal and professional roles are inseparable. Nurses need to be better prepared for the particular nursing practice they will encounter by way of appropriate training programmes (within acute medical care, preventive care, social work, psychology and pedagogy; but also information technology), to equip them to implement the ideology of health strategies.

This is an early exploratory study, and more research is required to further develop its findings; for example, by involving more nurses practicing in rural and remote Greenland to get a more complete picture of the necessary content for a systematic training programme that could become a pre-requisite for employment as a nurse in remote areas. Also, the viability of introducing a mobile clinical nurse, with academic competence, to support and supervise nurses in rural and remote Greenland requires investigation.

## Footnotes

1 Due to restricted funding, it was only possible to carry out fieldwork in four healthcare units and four nursing stations in rural and remote areas. The infrastructure of Greenland makes it very expensive to travel between towns and villages. We had the opportunity to travel to places on the coast at South Greenland, East Greenland and two places north of the capital Nuuk during 2011–2012.

2 Healthcare units are placed in towns in a Centre for Healthcare at the coast and the nurses have access to the interdisciplinary team, whereas nursing stations are placed in remote villages and the nurse is typically working only together with a health-assistant.
